# Editorial: Novel Molecular Targets for the Treatment of Pain

**DOI:** 10.3389/fnmol.2020.625714

**Published:** 2020-12-11

**Authors:** Tally M. Largent-Milnes, Meritxell Canals, John M. Streicher

**Affiliations:** ^1^Department of Pharmacology, College of Medicine, University of Arizona, Tucson, AZ, United States; ^2^Division of Physiology, Pharmacology and Neuroscience, School of Life Sciences, Queen's Medical Centre, University of Nottingham, Nottingham, United Kingdom; ^3^Centre of Membrane Proteins and Receptors, Universities of Birmingham and Nottingham, The Midlands, United Kingdom

**Keywords:** delta opioid receptor, mu opioid receptor, TIMP-1, heat shock protein 90, inflammation, cannabinoid, opioid, chronic pain

The chronic pain and opioid epidemics are two interdependent public health crises that have challenged the United States, in particular, and the world in general for more than 20 years. Chronic pain affects more than 100 million people in the USA, is growing in incidence as the population ages, can severely impact patient quality of life, and has economic costs of more than $600 billion in the USA alone (Breivik et al., 2009; Gaskin and Richard, 2012). In response, opioid prescribing has risen rapidly for two decades, resulting in an opioid abuse and overdose crisis that claims more than 40,000 lives annually in the USA (Lozano et al., 2012; Warner et al., 2016). These twin crises highlight the vast medical and social need to develop new treatments for chronic pain that are non-opioid or mitigate the negative effects of opioid therapy. However, despite a rapid increase in our understanding of the basic science of the pain and opioid systems, this knowledge has not yet translated into new therapies (Woodcock et al., 2007; Olson et al., 2017).

By identifying new targets and new approaches to treat pain, we may be able to design new therapies to efficaciously treat chronic pain without the drawbacks of current opioid therapies. This collection of novel basic science research articles titled “Novel Molecular Targets for the Treatment of Pain” is intended to stimulate research into these novel targets by the scientific community, which could then lead to the clinical development of new drugs.

This collection naturally falls into several themes, the first of which is novel regulation of the mu opioid receptor (MOR), the primary target of clinical opioids like morphine (Matthes et al., 1996; Olson et al., 2019). Original research from the Briddon and Canals groups demonstrated novel molecular events for the MOR after stimulation by the high efficacy agonist DAMGO; understanding of exactly how the MOR desensitizes and internalizes could lead to new methods to manipulate this process to improve opioid therapy (Gondin et al.). Several review articles also highlighted new areas of research into MOR regulation. The Filizola group reviewed recent advances in modeling the MOR activation process using molecular dynamics simulation (Ribeiro and Filizola). The Traynor group reviewed the role of Regulator of G Protein Signaling (RGS) proteins in regulating MOR activation, including the use of novel inhibitors to produce opioid-sparing or enhancement of endogenous opioid activity (Senese et al.). Lastly, the Veldhuis group reviewed exciting recent advances in separating membrane from internalized receptor signaling, how internalized signaling contributes to pain states, and how these disparate signaling states can be targeted by location-biased drugs (Retamal et al.).

The next major theme consisted of targeting other anti-nociceptive receptor systems to achieve pain relief without the side effects of MOR stimulation, particularly addiction, and respiratory depression. The Massotte group used elegant peripherally-restricted knockout of the delta opioid receptor (DOR) to demonstrate that the β2-adrenergic agonist formoterol produced efficacious anti-nociception in a neuropathic pain model via peripheral DOR (Ceredig et al.). This finding suggests that formoterol could be re-purposed from its current use as an adrenergic agonist as a novel non-opioid analgesic. The DOR has been a target of great interest for some time due to its ability to produce anti-nociception without addiction or respiratory depression, especially in inflammatory pain states. An overview of the DOR in pain and how it can be targeted in the future was written by the Gendron group, with a special emphasis on DOR intracellular trafficking, which strongly impacts receptor competency to relieve pain (Quirion et al.). The DOR has been implicated in other uses as well, such as the treatment of migraine pain (Charles and Pradhan, 2016).

Another alternate receptor system of interest is the cannabinoid receptor type-1 (CB_1_R). The CB_1_R can also produce anti-nociception, and while it can have unwanted psychoactive side effects, these side effects do not rise to the severity of addiction and respiratory depression caused by opioids (Rabgay et al., 2020). The CB_1_R is also the main target of the phytocannabinoid Δ9-tetrahydrocannabinol from the plant *Cannabis sativa*, and is thus of great interest considering the growth in recreational and medicinal marijuana (Morales et al., 2017). A study in this collection from the Laprairie group found that the non-steroidal anti-inflammatory drug indomethacin acts as a positive allosteric modulator of the CB_1_R (Laprairie et al.). This finding establishes a new CB_1_R drug scaffold for the creation of novel therapeutics.

The third theme of our collection is inflammatory regulation, which can contribute to both pain and the side effects of opioid drugs (Okun et al., 2011; Pan et al., 2016). Original research from the Schmidt group showed that granulocyte-colony stimulating factor (G-CSF) induced the recruitment of Ly6G positive neutrophils to the site of oral cancer, which released endogenous opioids to prevent or block oral cancer pain (Scheff et al.). This suggests that G-CSF could be used as a novel therapeutic for oral cancers. Work from the Jang-Hern Lee group showed that interleukin-1β was released in the early stages of spinal cord neuropathic pain to repress P450c17 expression and slow the development of neuropathic pain (Choi et al.), suggesting that enhancing activity of this pathway could slow or prevent the development of neuropathy. Lastly, the Laumet group provided a comprehensive review of the role of T cells in pain, including in the transition to chronic pain and resolution of pain, suggesting new ways to manipulate these cells to improve different pain states (Laumet et al.).

The last theme of our collection is higher level organization of proteins that contribute to pain and anti-nociception. The Baumbauer group provided original research showing that Tissue Inhibitor of Metalloproteinases-1 (TIMP-1) attenuates the development of inflammatory pain, by preventing the tissue remodeling performed by metalloproteinases (Knight et al.). This work suggests that new approaches to block MMP activity could block the development of pain states. The Streicher group also provided original research showing that the Heat shock protein 90 (Hsp90) isoform Hsp90α and the co-chaperones p23 and Cdc37 promote opioid anti-nociception in the brain (Lei et al.), highlighting the potential of isoform-selective Hsp90 inhibitors to improve opioid therapy.

Together, this collection highlights exciting new advances in the pain field using molecular neuropharmacology. The work of decades has made it clear that no one “silver bullet” is the key to solving the problem of the chronic pain and opioid crises. Multiple novel approaches from multiple angles, including the novel targets highlighted here, will be needed to construct a comprehensive and multi-targeted solution for these challenges and assist the most patients possible. Any of the targets highlighted in this collection could be exploited to create new therapeutics. Our goal is for this collection to contribute to that conversation, progress, and clinical advance to the benefit of society.

## Author Contributions

All authors contributed equally to the writing and editing of the manuscript.

## Conflict of Interest

The authors declare that the research was conducted in the absence of any commercial or financial relationships that could be construed as a potential conflict of interest.
